# Modulating the magnetic properties of MoS_2_ monolayers by group VIII doping and vacancy engineering†

**DOI:** 10.1039/c8ra01644e

**Published:** 2018-05-23

**Authors:** Cuifang Jia, Bo Zhou, Qi Song, Xiaodong Zhang, Zhenyi Jiang

**Affiliations:** Institute of Modern Physics, Shaanxi Key Laboratory for Theoretical Physics Frontiers, Northwest University Xi'an 710069 People's Republic of China zhoubo@nwu.edu.cn

## Abstract

In this work, density functional theory is adopted to study the electronic and magnetic properties of MoS_2_ monolayers combined with a single S vacancy defect and a group VIII (G8) atom dopant, in which the dopant is incorporated *via* Mo substitution. The calculated results show that the magnetic properties of monolayer MoS_2_ can be tuned by changing the distribution of the G8 atom and S vacancy. The S vacancy tends to decrease the net magnetic moment of the doped system when these two defects are in their closest configuration. By adjusting the distance between the dopant and the S vacancy, the doped MoS_2_ monolayer may show a variable net magnetic moment. In particular, all of the Ni-doped MoS_2_ monolayers show zero magnetic moment with or without an S vacancy. The mean-field approximation is used to estimate the Curie temperature (*T*_C_). Our results show that Fe, Co, Ru, Rh, Os and Ir-doped MoS_2_ monolayers are potential candidates for ferromagnetism above room temperature. The density of states calculations provide further explanations as to the magnetic behavior of these doped systems. These results provide a new route for the potential application of atomically thin dilute magnetic semiconductors in spintronic devices by employing monolayer MoS_2_.

## Abstract 2

In this work, the density functional theory study shows that the magnetic properties of MoS_2_ monolayer can be tuned by the distribution of group VIII atom and S vacancy, in which the dopant is incorporated *via* Mo subsitution.

## Graphical abstract

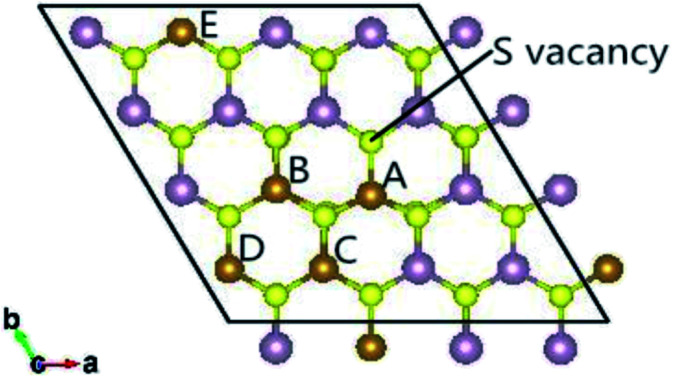

## Introduction

Dilute magnetic semiconductors (DMSs) have been the focus of extensive research over the last decade, driven by the prospect of achieving spintronic devices, which can exploit both the charge and spin freedom.^1–5^ Meanwhile, two-dimensional (2D) transition metal dichalcogenides (TMDCs) have also demonstrated great potential for the new generation spintronics devices due to their unique structural and electronic properties. A significant amount of theoretical and experimental effort has been devoted in the field of TMDCs to comprehending the role of magnetic impurities such as V, Cr, Mn, Fe, Co, Ni, and Cu as discussed in several reviews.^5–10^ Despite the great advances in this field, producing strong ferromagnetic interactions and the stability of such ferromagnetic order is still the most important and most difficult part.

Recently, many studies, theoretical and experimental, have focused on the magnetic properties of 1H-MoS_2_.^11–13^ Magnetic doping through either adsorption or substitution is found to be an efficient method to introduce magnetism into MoS_2_.^5–7^ Magnetic interactions can be tuned by carriers and strain. At the same time, sulfur vacancies have also been found to be related to the magnetic properties of MoS_2_.^14^ Vacancies not only influence the magnetic properties, but also change the carrier density of the semiconductor. Obviously, S vacancies can influence the magnetism in the transition metal doping case. Lots of studies have been done on each factor. However, it is not clear how these two factors interplay and then what the influence of this interplay is on the magnetic properties.

In this paper, theoretical methods are used to study the effect of substitutional group VIII (G8) atom doping in 1H-MoS_2_ with or without an S vacancy. The distributions of the S vacancy and G8 elements have also been considered. This work may bring a new insight into the preparation of ferromagnetic materials in DMSs.

## Computational methods

First principles calculations were performed using the Vienna *ab initio* simulation package (VASP)^15^ on the basis of density functional theory (DFT). The electron–ion interactions were described by the projected augmented wave (PAW) method,^16^ and the electronic exchange–correlation potential employed the generalized gradient approximation with the Perdew–Burke–Ernzerhof functional (GGA-PBE).^17^ In order to verify our results, the Hubbard-*U* correction method^18^ was also used. Different *U* values were assigned to the G8 impurities, while the *U* parameterization was not used for the host materials since they have little impact on the magnetic ordering, as suggested by many authors in ref. 19–21. A 4 × 4 × 1 MoS_2_ supercell structure containing 32 S and 16 Mo atoms was constructed as the pristine model in this calculation. Moreover, a vacuum region of 15 Å was added along the *c* plane to minimize the interaction between the adjacent periodic images. The cutoff energy for the plane-wave expansion is set at 500 eV after extensive convergence analysis. The Brillouin zone (BZ) is sampled using a 3 × 3 × 1 gamma-centered Monkhorst–Pack grid. The valence electron configurations in this calculation are Mo 4p^6^ 4d^5^ 5s^1^, S 3s^2^ 3p^4^, Fe 3d^6^ 4s^2^, Co 3d^7^ 4s^2^, Ni 3d^8^ 4s^2^, Ru 4d^7^ 5s^1^, Rh 4d^8^ 5s^1^, Pd 4d^10^, Os 5d^6^ 6s^2^, Ir 5d^7^ 6s^2^, and Pt 5d^8^ 6s^2^, respectively. All of the structures are fully relaxed using the conjugate gradient method, and during the structural relaxation, the energy convergent criterion is 10^−6^ eV per unit cell, and the forces on all relaxed atoms are less than 0.02 eV Å^−1^.

## Results and discussion

### A. Tendency of the formation energy

**Fig. 1:**
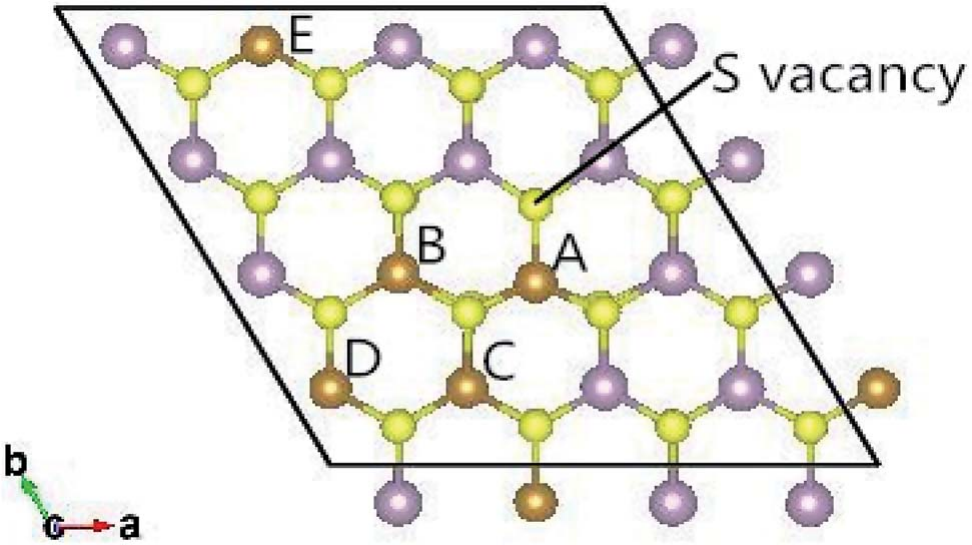
Top view of the 4 × 4 × 1 supercell of the defective 1H-MoS_2_ impurities. Five configurations are taken into account in this work and marked as Mo_15_XS_31_-A, B, C, D, or E according to the distance between the dopant and S vacancy.

The fully relaxed lattice constant of monolayer MoS_2_ is 3.19 Å and the Mo–S bond length is 2.42 Å, which agrees with the experimental value (3.16 Å).^22^ Defects play an important role in the electronic and magnetic properties of materials. Doping atoms or vacancies in 1H-MoS_2_ are introduced by replacing or removing a single host atom in the pristine system. In this paper, we mainly consider the G8 atoms as impurities to substitute a host Mo atom in both host materials. For the doped pristine 1H-MoS_2_, the Mo atoms are replaced by impurity atoms, represented as Mo_15_XS_32_ (X = Fe, Co, Ni, Ru, Rh, Pd, Os, Ir, or Pt). Structure relaxation shows that the distances between the 3d atoms (Fe, Co, and Ni) and the adjacent three S atoms are roughly similar and slightly shorter than the original Mo–S bond length. For the 4d and 5d atoms, the larger the number of extranuclear electrons, the longer the bond length. For the doped defective 1H-MoS_2_, the configurations are marked as Mo_15_XS_31_-A, B, C, D, or E according to the distance between the dopant and the S vacancy, as shown in Fig. 1. The maximum displacement of the dopant and the S vacancy is 7.53 Å (pristine Mo_15_XS_31_-D) and the minimum is 2.42 Å (pristine Mo_15_XS_31_-A). This corresponds to simulating a Mo_1−*x*_A_*x*_S_2−*y*_ periodic system with *x* = 6.25% (*y* = 3.13%). Furthermore, to characterize the deviation of the impurity atom from the original Mo position, we calculated the root-mean-square deviation (RMSD) of the X–S bond length using the following formula:1
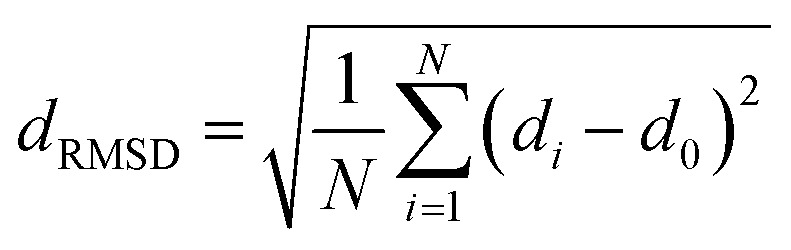
where *N* represents the number of X–S bonds around the dopants. The value of *N* is 6, except for the nearest neighboring structures which have five X–S bonds. *d*_0_ is the Mo–S length in pristine MoS_2_, while *d*_*i*_ is the X–S bond length in the doped model. The results are listed in Table 1.

**Table 1:** Optimized parameters for the G8-doped pristine and defective 1H-MoS_2_ systems Mo_1−*x*_A_*x*_S_2−*y*_ with *x* = 6.25% (*y* = 3.13%). The root-mean-square deviation (RMSD) of the X–S bond length (*d*_RMSD_ in Å), the formation energy (*E*_form_ in eV), the total magnetic moments of the PBE results (*M*_tot_ in *μ*_B_), the Bader charge (*Q* in e) of the G8 atom, and the energy band gap (*E*_g_ in eV)

Doped-atom	Configuration	*d*_RMSD_ (Å)	*E*_form_ (eV)		*M*_tot_ (*μ*_B_)	*Q* (e)	*E*_g_ (eV)
			Mo-rich	S-rich			
Fe	Mo_15_FeS_32_	0.121	2.64	−0.06	2.00	6.95	0.30
	Mo_15_FeS_31_-A	0.152	6.61	5.26	0	7.11	0.33
	Mo_15_FeS_31_-B	0.117	7.79	6.44	2.00	6.99	0.19
	Mo_15_FeS_31_-C	0.113	7.80	6.45	2.00	7.00	0.27
	Mo_15_FeS_31_-D	0.118	7.76	6.41	2.00	6.98	0.37
	Mo_15_FeS_31_-E	0.119	7.82	6.47	2.00	6.98	0.27
Co	Mo_15_CoS_32_	0.120	3.91	1.21	3.00	8.23	0.18
	Mo_15_CoS_31_-A	0.169	7.19	5.84	1.00	8.32	0.21
	Mo_15_CoS_31_-B	0.122	9.05	7.70	3.00	8.21	0.24
	Mo_15_CoS_31_-C	0.444	8.66	7.29	1.00	8.29	0.28
	Mo_15_CoS_31_-D	0.134	8.97	7.68	1.00	8.24	0.22
	Mo_15_CoS_31_-E	0.118	9.09	7.74	3.00	8.22	0.11
Ni	Mo_15_NiS_32_	0.029	4.72	2.22	0	9.35	0.28
	Mo_15_NiS_31_-A	0.175	7.82	6.47	0	9.37	0.43
	Mo_15_NiS_31_-B	0.034	9.75	8.74	0	9.37	0.15
	Mo_15_NiS_31_-C	0.030	9.57	8.57	0	9.36	0.30
	Mo_15_NiS_31_-D	0.411	9.70	8.35	0	9.35	0.27
	Mo_15_NiS_31_-E	0.031	9.88	8.74	0	9.35	0.27
Ru	Mo_15_RuS_32_	0.032	3.05	0.35	2.00	7.14	0.17
	Mo_15_RuS_31_-A	0.067	6.54	5.19	0	7.32	0.57
	Mo_15_RuS_31_-B	0.039	7.90	6.60	0	7.19	0.34
	Mo_15_RuS_31_-C	0.037	7.96	6.61	0	7.20	0.26
	Mo_15_RuS_31_-D	0.046	7.92	6.57	0	7.18	0.38
	Mo_15_RuS_31_-E	0.048	8.04	6.69	0	7.18	0.23
Rh	Mo_15_RhS_32_	0.033	4.17	1.47	1.00	8.39	0.03
	Mo_15_RhS_31_-A	0.060	7.22	5.87	1.00	8.52	0.19
	Mo_15_RhS_31_-B	0.293	8.98	7.63	1.00	8.47	0.28
	Mo_15_RhS_31_-C	0.415	8.72	7.23	1.00	8.49	0.20
	Mo_15_RhS_31_-D	0.038	9.09	7.74	1.00	8.40	0.15
	Mo_15_RhS_31_-E	0.035	9.21	7.86	1.00	8.41	0.10
Pd	Mo_15_PdS_32_	0.298	5.03	2.33	0	9.58	0.30
	Mo_15_PdS_31_-A	0.058	8.28	6.93	0	9.60	0.49
	Mo_15_PdS_31_-B	0.347	10.08	8.73	2.00	9.59	0.19
	Mo_15_PdS_31_-C	0.355	9.86	8.51	0	9.59	0.42
	Mo_15_PdS_31_-D	0.356	10.08	8.73	0	9.58	0.28
	Mo_15_PdS_31_-E	0.358	10.12	8.77	0	9.57	0.27
Os	Mo_15_OsS_32_	0.031	3.59	0.89	2.00	7.06	0.12
	Mo_15_OsS_31_-A	0.065	7.08	5.73	0	7.30	0.58
	Mo_15_OsS_31_-B	0.035	8.26	6.91	0	7.14	0.40
	Mo_15_OsS_31_-C	0.026	8.43	7.08	2.00	7.15	0.21
	Mo_15_OsS_31_-D	0.043	8.36	7.01	0	7.11	0.35
	Mo_15_OsS_31_-E	0.044	8.50	7.15	0	7.11	0.16
Ir	Mo_15_IrS_32_	0.032	4.38	1.68	1.00	8.44	—
	Mo_15_IrS_31_-A	0.059	7.46	6.11	1.00	8.60	0.23
	Mo_15_IrS_31_-B	0.048	9.21	7.86	1.00	8.48	0.35
	Mo_15_IrS_31_-C	0.032	9.19	7.84	1.00	8.50	0.19
	Mo_15_IrS_31_-D	0.038	9.22	7.87	1.00	8.46	0.11
	Mo_15_IrS_31_-E	0.040	9.36	8.01	1.00	8.47	0.09
Pt	Mo_15_PtS_32_	0.322	4.98	2.28	0	9.68	0.32
	Mo_15_PtS_31_-A	0.055	8.14	6.79	0	9.72	0.58
	Mo_15_PtS_31_-B	0.373	9.85	8.50	0	9.70	0.36
	Mo_15_PtS_31_-C	0.372	9.77	8.42	0	9.71	0.45
	Mo_15_PtS_31_-D	0.392	9.93	8.58	0	9.68	0.27
	Mo_15_PtS_31_-E	0.393	9.95	8.60	0	9.67	0.28

In order to inspect the stability and feasibility of all optimized geometrical structures, the formation energies were obtained utilizing the following formula:^23–25^

2 *E*_form_ = *E*_doped_ − *E*_pure_ + (*μ*_Mo_ − *μ*_G8_) + *μ*_S_

where *μ*_doped_ and *E*_pure_ represent the total energies of the G8-doped monolayer MoS_2_ and pure one. *μ*_Mo_, *μ*_S_ and *μ*_G8_ are the chemical potentials for the Mo host, S host and G8 dopant atoms, respectively. All of the chemical potentials of the G8 elements are obtained from faced-centered cubic (fcc) structures except for Fe, which uses a body-centered cubic (bcc) structure. The formation energy of MoS_2_ itself, *E*_form_(MoS_2_), can be calculated from the expression:

3 *E*_form_(MoS_2_) = *μ*_MoS_2__ − *μ*^0^_Mo_ − 2*μ*^0^_S_

where *μ*_MoS_2__ is equal to *E*_pure_ per MoS_2_ formula unit, and *μ*^0^_Mo_/*μ*^0^_S_ is the total energy per atom of Mo/S in its reference phase. For Mo, the reference phase is the bulk bcc metal. The reference phase for S is the S8 ring, which is the most stable state at room temperature. Thus, the value of *E*_form_(MoS_2_) is −2.70 eV in DFT-GGA, consistent with the value of −2.84 eV reported in ref. 26.

The values of *μ*_Mo_ and *μ*_S_ in eqn (2) depend on the experimental growth conditions. For the Mo-rich case, the Mo chemical potential is equal to the bulk Mo value, *μ*^Mo-rich^_Mo_ = *μ*^0^_Mo_, and the S chemical potential can be obtained from *μ*_MoS_2__ = *μ*_Mo_ + 2*μ*_S_ on the basis of thermodynamic equilibrium. Hence, combined with eqn (3), the chemical potentials for the Mo-rich limit can then be written as:

4 *μ*^Mo-rich^_Mo_ = *μ*^0^_Mo_,

5
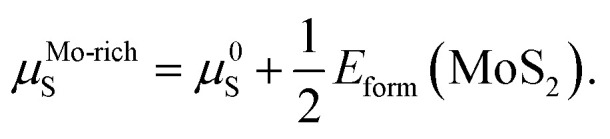


Likewise, under S-rich conditions, the values are:

6 *μ*^S-rich^_Mo_ = *μ*^0^_Mo_ + *E*_form_(MoS_2_),

7 *μ*^S-rich^_S_ = *μ*^0^_S_.

All of the formation energies are listed in Table 1.

**Fig. 2:**
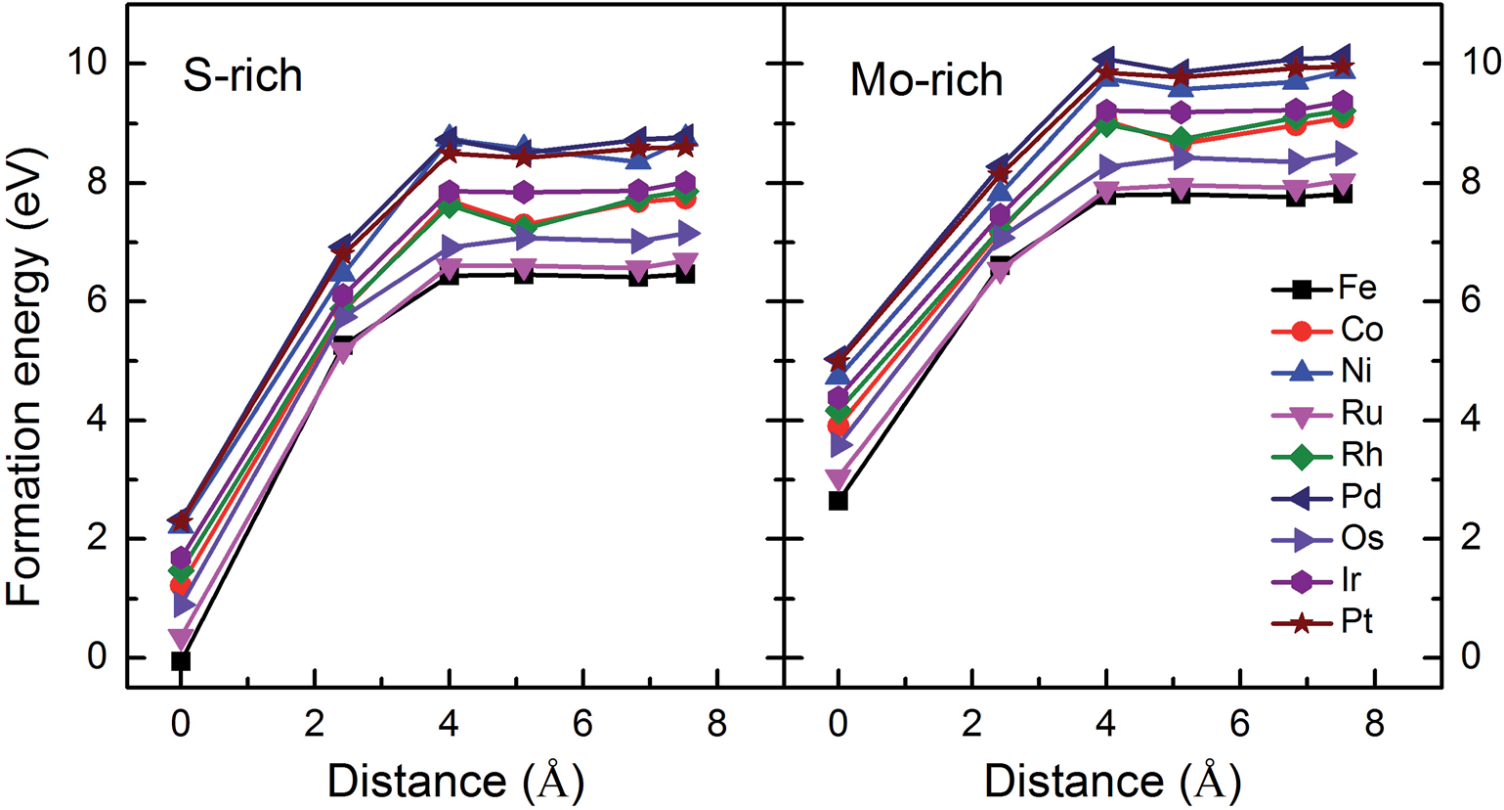
Formation energies as a function of the distance between the S vacancy and impurity atoms in S-rich and Mo-rich conditions.

Monolayer MoS_2_ with a single S vacancy (V_S_) or Mo vacancy (V_Mo_) was fully relaxed. The optimized structure shows that the neighboring Mo and S atoms have slight displacements with respect to the vacancy site V_S_ and V_Mo_, which is different from the obvious reconstruction in a graphene sheet with a single C vacancy.^27^ For V_S_-MoS_2_, this is more likely to occur under S-rich conditions and the formation energy is 6.57 eV, which in agreement with the previous values of 5.89 (ref. 28) and 5.72 eV,^7^ is much lower than the formation energy of the Mo vacancy V_Mo_ (14.09 eV) in Mo-rich conditions. The previous studies have reported that the substitution of an Mo site is more stable than that of an S site.^7,29^ Experimentally, S vacancies are more common than Mo vacancies.^30^ Therefore, in this paper, the co-doped configurations mainly consist of an S vacancy and G8 impurity substitution of an Mo site. The *C*_3v_ symmetry of pristine monolayer MoS_2_ is destroyed after G8 element doping, and the distances between the impurities and the nearest S atom change with different distributions of the dopants and the vacancy. All the calculated formation energies are summarized in Table 1. The distances between the dopants atoms and the S vacancy are taken from their original positions in pristine 1H-MoS_2_. Fig. 2 shows our calculated formation energies as a function of the distance between the S vacancy and dopant atoms, which varies from 2.42 to 7.53 Å. The first data “0” represents the doped MoS_2_ without an S vacancy. According to our theoretical results, Fe doping is most favorable energetically among all considered impurities. For all of the G8 elements, the formation energies from the doped defective 1H-MoS_2_ configurations of the second-, third-, fourth-, and fifth-nearest neighbor are very close, and are 1.50 eV larger than the corresponding nearest neighboring case. The relevant data for the Co atom are consistent with ref. 31.

The positive formation energy indicates that the formation of the vacancy defect and the doping of the transition metal are endothermic processes. The equilibrium concentrations of the vacancies are usually very low because of their high formation energies. Nonetheless, new techniques have been developed to create these defects. The generation of nanomesh size vacancies in graphene has been reported.^32,33^ Vacancy engineering of a doped MoS_2_ monolayer can also be achieved.

### B. Magnetic properties

**Fig. 3:**
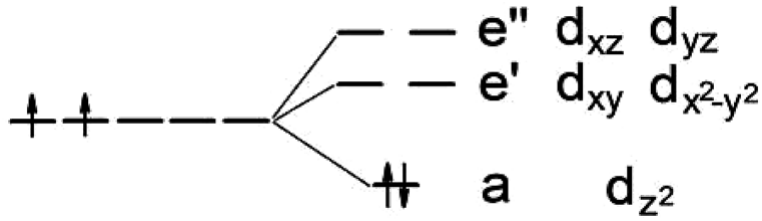
Ligand field picture and corresponding d-orbitals for a trigonal prismatic coordination (*D*_3h_ symmetry) in a d-metal dichalcogenide with a d^2^ configuration of the metal atom.

It is known that MoS_2_ has *D*_3h_ symmetry and the d-orbitals have a schematic band structure with a d^2^ configuration of the metal atom, as shown in Fig. 3. The two valence electrons of the Mo ion occupy the lowest d_*z*^2^_ orbital, which is the reason for the lack of magnetism for MoS_2_. In our case, the sulfur atom vacancy introduces two defective bands in the band gap region, which mainly consists of the “unsaturated” d orbitals of Mo atoms. The relatively large energy gap of MoS_2_ can almost accommodate the five d states of TM magnetic impurities. Therefore, about seven defective bands may appear in the forbidden gap region of the pristine bands when the doped G8 atoms are incorporated with an S vacancy. The magnetic properties mainly depend on the correlation between these defective bands.

The Fe case, which corresponds to the d^4^ configuration, is likely to have a net magnetic moment of 2*μ*_B_, except for the A case. In the nearest configuration, the interaction between the Fe and the S vacancy (the unsaturated Mo atoms) leads to strong crystal field splitting between the d_*z*^2^_, d_*xy*_, and d_*x*^2^−*y*^2^_ orbitals, which causes the system to become a small band gap semiconductor. Among all of the five pristine dopant-vacancy configurations, the B and E cases have more symmetry and the vertical mirror plane is preserved. It is important for the system to keep the energy levels of the hybridized states below the Fermi level in the close energy range. This can explain the net magnetic moment of the B and E cases of Fe, and Co. On the other side, the C and D cases are more likely to have zero magnetic moment. Our results also indicate that local stress appears to be a crucial factor in the development of magnetism as Jahn–Teller distortions that destroy the *C*_3v_ lattice symmetry lead to the disappearance of magnetism. For the Fe and Co cases, the C and D configurations may also have a net magnetic moment because of the coupling between the defective d orbital of the unsaturated Mo atoms and the lower d_*xy*_, d_*x*^2^−*y*^2^_, and d_*z*^2^_ orbitals of the impurity atom.

For 4d or 5d dopants such as the Ru-doped cases, only the structure of mono-doped Mo_15_RuS_31_ generates a 2*μ*_B_ magnetic moment. The origin of the magnetism is attributed to the near degeneracy of the Ru d_*x*^2^−*y*^2^_ and d_*z*^2^_ orbitals. The Os-doping has similar characteristics to the Ru doped cases. For the Pd-doping, only the Mo_15_PdS_31_-B structure generates a 2*μ*_B_ magnetic moment. For the Rh- and Ir-doping, all of the structures generate a 1*μ*_B_ magnetic moment. For the Pt-doping structures, all are nonmagnetic. Taken as a whole, the net magnetic moments from the 3d elements are larger than those from the 4d and 5d elements, because strongly localized 3d orbitals are more likely to induce net magnetic moments due to strong Hund coupling, which competes with the ligand field energy splitting.

Furthermore, the ligand field energy splitting is connected with the distortion of the local structure around the dopants. The root-mean-square deviations of the X–S bonds have been obtained to quantify the local deformation caused by the dopants and are listed in Table 1. The results clearly show that the RMSDs of the models with smaller magnetic moments are much larger, which means a larger deviation from *C*_3v_ symmetry. This rule holds for the Fe, Co and Os cases.

To verify our results, the DFT+U method was also used. It was found that the magnetic properties with the GGA+U method are consistent with the results without the Hubbard-*U* parameter. The results with the corresponding *U* values are shown in ESI Table S1.† There is no relevant influence on our conclusions and therefore we discuss in the following the results without on-site interaction.

**Fig. 4:**
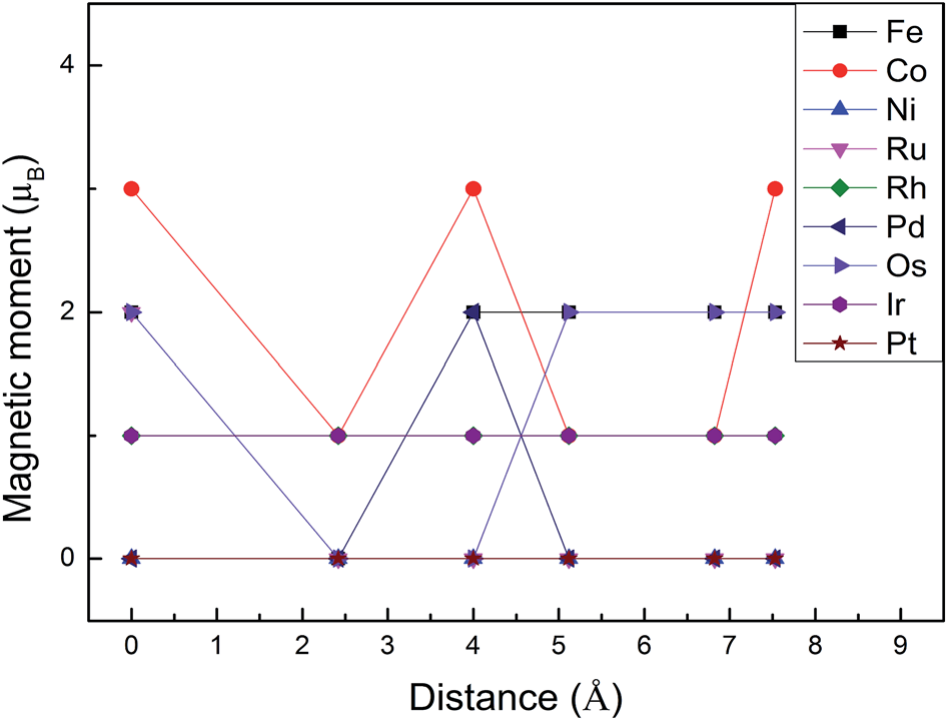
Summary of the magnetic moments for all G8-doped 1H-MoS_2_ systems.

The system is stabilized by charge transfer. The Bader charge has also been obtained to analyze the charge transfer between the G8 atoms and MoS_2_, as shown in Table 1. In a perfect 1H-MoS_2_, the formal valences of Mo and S are +4 and −2, respectively. Since a Mo has six S neighbors, it contributes 2/3 electrons to each Mo–S covalent bond. Therefore, the charge transfer of the S and Mo atoms is approximately +0.67e and −1.33e, respectively. Taking Fe-doped 1H-MoS_2_ as an example, in the monodoping case, the charge number of Fe will be 8 − 2 × 2/3 = 6.67 (8 is the valence electron number of Fe treated by the PBE pseudopotential). It will be 8 − 5/3 × 2/3 = 6.89 when one S atom is removed. The theoretical charge numbers of Ru and Os are equal to that of Fe due to their similar valence electron numbers. For the other G8 elements, the two values for mono- and co-doping are 7.67 and 7.89 for Co, Rh and Ir, and 8.67 and 8.89 for Ni, Pd and Pt. The more the Bader charge deviates from the ideal charge number, the more electron transfer there is from the host ions to the dopant ion (Fig. 4).

**Fig. 5:**
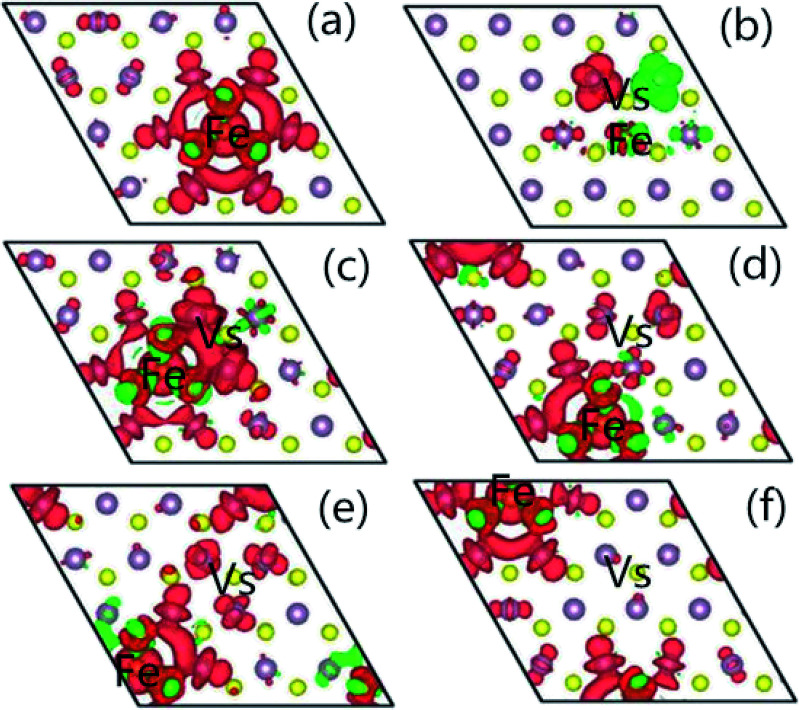
The spatial distribution of the spin density for (a) Mo_15_FeS_32_, (b) Mo_15_FeS_31_-A, (c) Mo_15_FeS_31_-B, (d) Mo_15_FeS_31_-C, (e) Mo_15_FeS_31_-D, and (f) Mo_15_FeS_31_-E. The S, Mo, and Fe atoms are denoted by yellow, purple, and dark yellow spheres, respectively. The red isosurface corresponds to the spin-up density, and the green one represents the spin-down density.

In order to understand the characteristics of the magnetic moment induced by impurity states in the G8-doped 1H-MoS_2_ systems, the spin density distribution is plotted to visualize the distribution of the magnetic moments of the doped 1H-MoS_2_ systems in Fig. 5. Taking Fe-doped 1H-MoS_2_ as an example, Fig. 5(a)–(f) show that the spatial extensions of the spin polarizations have reached the first-nearest S atoms and the second-nearest Mo atoms. The structure Mo_15_FeS_32_ displays weak ferromagnetic and antiferromagnetic coupling between Fe and three neighboring S atoms, and ferromagnetic coupling among the six second-nearest Mo atoms. Fig. 5(b) shows the nearest configuration of the co-doping system, which is typical for the no magnetism case. The antiferromagnetic alignment of d electrons is observed.

### C. Estimation of the Curie temperature

The Curie temperature (*T*_C_) values can be roughly estimated from the mean-field expression^34^ using the relation 
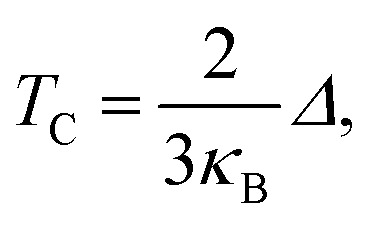
 where *κ*_B_ is the Boltzmann constant and *Δ* is the difference in the supercell total energy between the antiparallel and parallel alignments. The results are listed in Table 2. Our theoretical results show that Fe, Co, Ru, Rh, Os and Ir-doped MoS_2_ are candidates for room-temperature ferromagnetic materials in DMSs. It is should also be noticed that the mean field approximation used in this paper is known to overestimate the Curie temperature.^35^ Nevertheless, a high *Δ* will imply a high *T*_C_ and the trends presented here should hold.

**Table 2:** The spin-flip energies (*Δ* in eV) and estimated Curie temperatures (*T*_C_ in Kelvin) for all of the configurations that have net spin moments

		Mo_15_XS_32_	Mo_15_XS_31_-A	Mo_15_XS_31_-B	Mo_15_XS_31_-C	Mo_15_XS_31_-D	Mo_15_XS_31_-E
Fe	*Δ*	0.209	—	0.138	0.037	0.053	0.175
	*T*_C_	1614	—	1106	284	410	1352
Co	*Δ*	0.110	0.060	0.114	0.072	0.060	0.027
	*T*_C_	850	462	882	556	464	208
Ru	*Δ*	0.125	—	—	—	—	—
	*T*_C_	966	—	—	—	—	—
Rh	*Δ*	0.026	0.099	0.070	0.050	0.039	0.026
	*T*_C_	200	766	542	386	302	200
Pd	*Δ*	—	—	0.010	—	—	—
	*T*_C_	—	—	78	—	—	—
Os	*Δ*	0.088	—	—	0.032	—	—
	*T*_C_	680	—	—	248	—	—
Ir	*Δ*	0.022	0.053	0.090	0.055	0.031	0.017
	*T*_C_	170	410	696	426	240	132

### D. Density of states

**Fig. 6:**
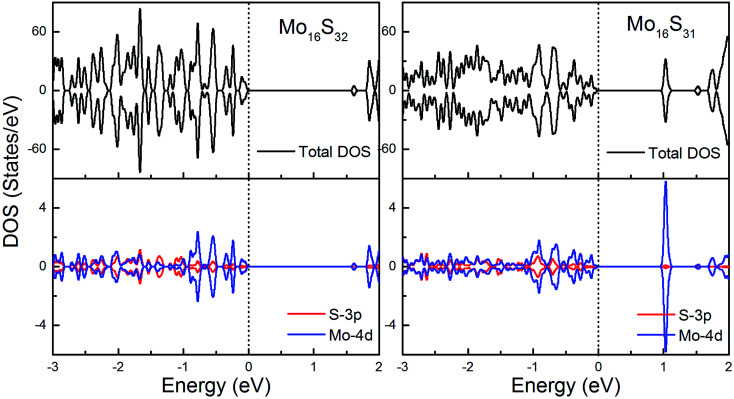
Total DOS and partial DOS for pristine 1H-MoS_2_ with and without S vacancies. The dashed line indicates the Fermi level at 0 eV.

To gain further insight into the emergent magnetic behavior, the total density of states (TDOS) and projected density of states (PDOS) of all of the models are shown, which also provides a specific description of the ground state electronic structure. The upper and lower panels denote the TDOS and PDOS, and positive and negative values represent spin-up and spin-down channels, respectively. The Fermi level is set at zero energy, which is indicated by black dotted lines, to easily identify the band gap and the relative position of the states from the impurity atoms. Fig. 6 shows the DOS and PDOS of pristine 1H-MoS_2_ and defective 1H-MoS_2_ with one S vacancy. We can see that both of them are nonmagnetic semiconductors with the spin-up and spin-down channels completely symmetrical, and the former opens a direct gap of 1.67 eV, agreeing with the experimental value (1.90 eV) as well as other calculation results (1.66 eV (ref. 31) and 1.67 eV (ref. 36)), while the latter shows an indirect band gap of 1.06 eV. After the removal of the S atom, new impurity states appear above the Fermi level, which are mainly from the 4d orbitals of Mo atoms.

**Fig. 7:**
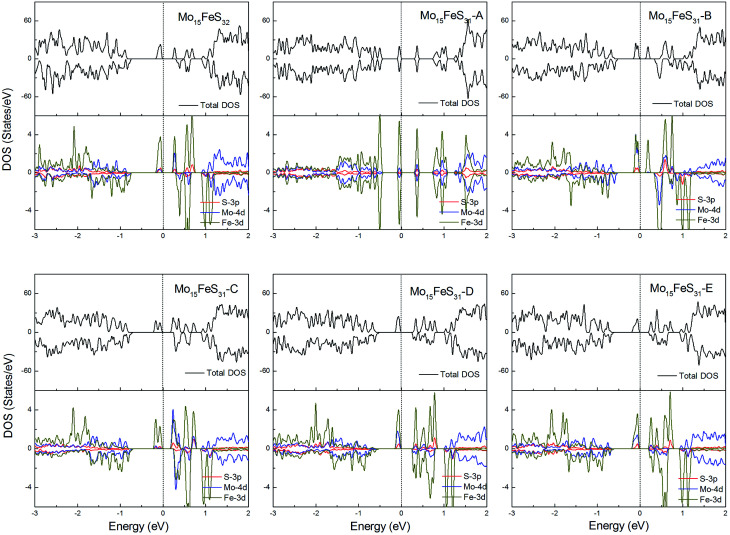
Total DOS and partial DOS for each atomic species in the all Fe-doped 1H-MoS_2_. The dashed line indicates the Fermi level at 0 eV.

The density of states of all the Fe-doped MoS_2_ models are shown in Fig. 7. It can be found that the impurity states presented in the band gap region are mainly contributed by the Mo 4d, Fe 3d, and S 3p orbitals. All of these systems are ferromagnetic with net magnetic moments of 2*μ*_B_, except for the structure Mo_15_FeS_31_-A, which is a nonmagnetic semiconductor with a 0.3 eV band gap. In configuration A, the largest deformation of the Fe atom can be found from visualizing the optimized structure, which results in the removal of the degeneracy of the 3d orbitals and leads to the formation of the localized hydride states near the valance bands. As for the five magnetic systems, as expected, the Fe dopant is a main contributor to the total magnetic moment. This feature applies to all G8 impurity-doped configurations in this calculation.

**Fig. 8:**
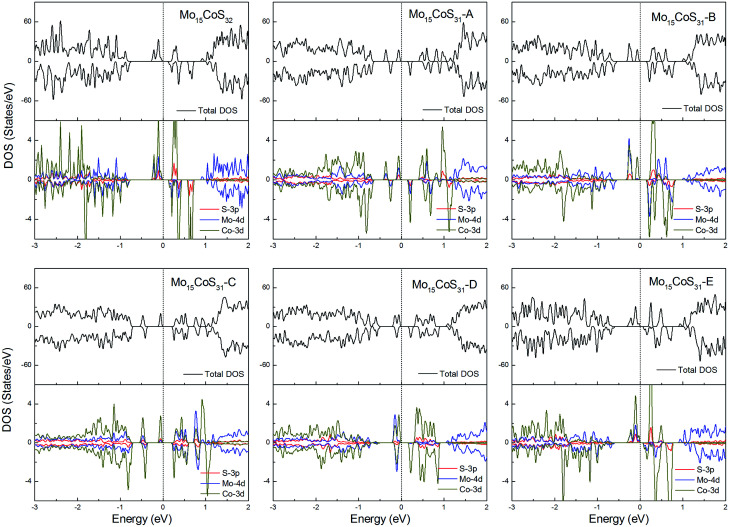
Total DOS and partial DOS for each atomic species in the all Co-doped 1H-MoS_2_.

For the Co-doped cases, these systems have odd electrons. They are all ferromagnetic with a magnetic moment of 3 or 1*μ*_B_. Unlike the Fe case, the correlation between the Co atom dopant and the S vacancy causes the impurity states to spread in the whole band gap region. Especially for the configurations Mo_15_CoS_31_-A and Mo_15_CoS_31_-D, the strong interaction between the Co–Mo–S pairs leads to the formation of a localized impurity state near the valance bands. The decrease in the magnetic moment can be attributed to the pairing of the electrons in this mixed orbital. In other words, the magnetic properties of the co-doped system depend on the competition between the ligand field splitting and the Hund coupling (Fig. 8).

**Fig. 9:**
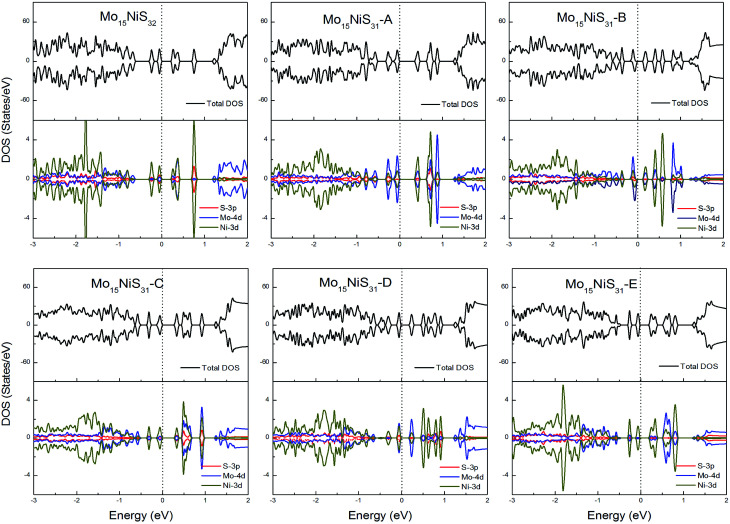
Total DOS and partial DOS for each atomic species in the all Ni-doped 1H-MoS_2_.

For Ni-doped 1H-MoS_2_ systems, there are 4 unpaired electrons remaining, which would lead to about a 4*μ*_B_ magnetic moment. But from our calculations, all of the ground states of the Ni-doped models are nonmagnetic semiconductors where the spin-up and spin-down channels are completely symmetrical and they possess tiny band gaps of 0.28, 0.43, 0.15, 0.30, 0.27, and 0.27 eV, respectively. The strong crystal field of neighboring Mo and S atoms leads to large energy splitting of the defective orbital. Therefore, four outer electrons form two electron pairs without the existence of isolated electrons. During our calculations, we found that the Mo_15_NiS_32_ and Mo_15_NiS_31_-B, C and E configurations would relax to transition states that have high magnetic order (net magnetic moment 4*μ*_B_). These transition states are local minima which are 199, 341, 355, and 208 meV above the global minima. In a recent experimental study,^9^ the author reported that 4% Ni doped MoS_2_ has a paramagnetic phase at room temperature, and the paramagnetic phase may dominate at low temperature. Our theoretical results of the transition states with an S vacancy provide an explanation for this phenomenon (Fig. 9).

**Fig. 10:**
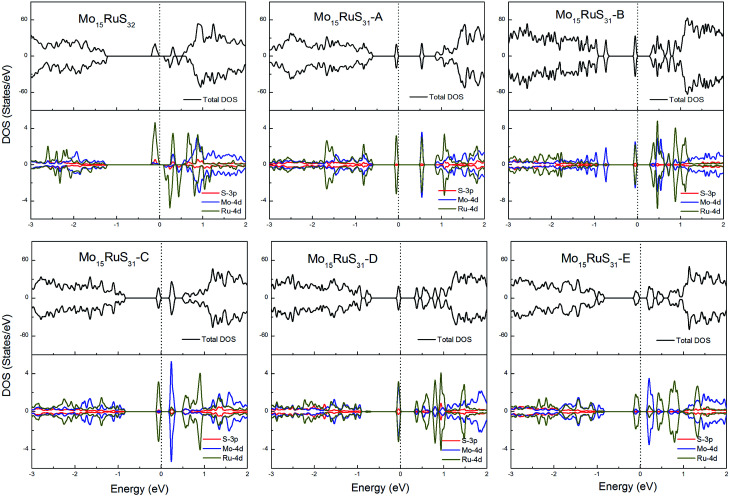
Total DOS and partial DOS for each atomic species in the all Ru-doped 1H-MoS_2_.

For the Ru-doped cases, substitution of Mo atoms by Ru increases the degree of p–d hybridization leading to a shift of the majority spin below the Fermi level. All configurations with the existence of S vacancies have semiconductor character with a band gap shown in Table 1, while the structure Mo_15_RuS_32_ is a magnetic semiconductor with a net magnetic moment of 2*μ*_B_. The magnetism of Mo_15_RuS_32_ can be attributed to the formation of degenerated bands mainly consisting of the Ru d_*x*^2^−y^2^_ and d_*z*^2^_, Mo 4d and S 3p orbitals (Fig. 10).

**Fig. 11:**
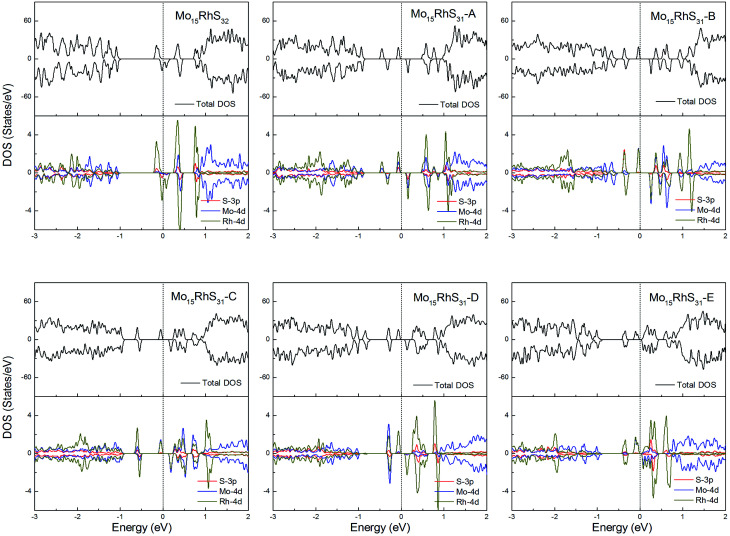
Total DOS and partial DOS for each atomic species in the all Rh-doped 1H-MoS_2_.

Rh-doping provides 3 more valence electrons than the host Mo atom and all structures generate a 1*μ*_B_ magnetic moment. In addition, the mono-doped structure is a half-metal and the others are magnetic semiconductors (Fig. 11).

**Fig. 12:**
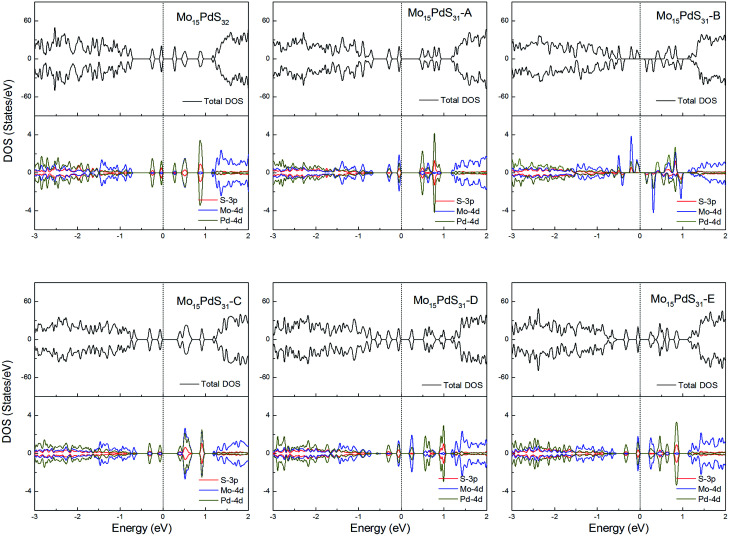
Total DOS and partial DOS for each atomic species in the all Pd-doped 1H-MoS_2_.

For Pd-doped 1H-MoS_2_, the structure Mo_15_PdS_31_-B is a magnetic semiconductor with a 2*μ*_B_ magnetic moment, although Pd is a nonmagnetic element. The other systems are all nonmagnetic semiconductors and generate tiny band gaps of 0.30, 0.58, 0.21, 0.35, and 0.16 eV for Mo_15_PdS_32_, Mo_15_PdS_31_-A, Mo_15_PdS_31_-C, Mo_15_PdS_31_-D, and Mo_15_PdS_31_-E, respectively. The reason for the magnetism is the Hund coupling between the two hybridized states from the Mo 4d, S 3p and Pd 4d orbitals (Fig. 12).

**Fig. 13:**
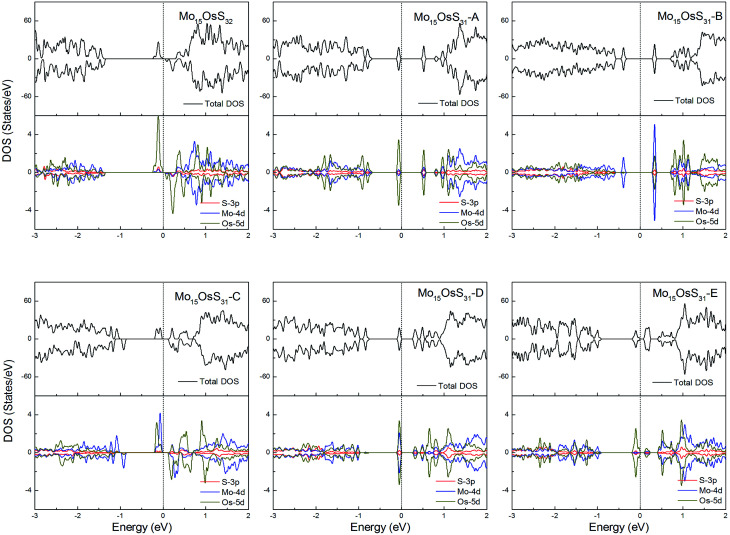
Total DOS and partial DOS for each atomic species in the all Os-doped 1H-MoS_2_.

Os is a 5d element with less localized d orbitals than the 3d and 4d elements discussed above. For the Os-doped 1H-MoS_2_, the impurity states are located near the conduction bands. The Mo_15_OsS_32_ and Mo_15_OsS_31_-C configurations are magnetic, and both have a net magnetic moment of 2 *μ*_B_. In the Mo_15_OsS_31_-C configuration, the magnetism is attributed to the near degeneracy of two defective bands composed of the Co d_*x*^2^−*y*^2^_ and d_*z*^2^_ orbitals and the d orbital of the nearby Mo ions. The Mo_15_OsS_31_-A, Mo_15_OsS_31_-B, Mo_15_OsS_31_-D and Mo_15_OsS_31_-E configurations are nonmagnetic semiconductors with small band gaps of 0.57, 0.40, 0.35 and 0.16, respectively (Fig. 13).

**Fig. 14:**
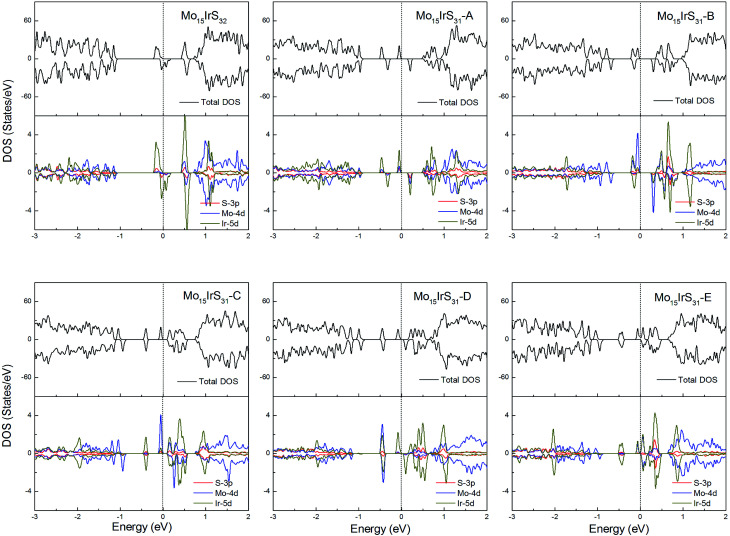
Total DOS and partial DOS for each atomic species in the all Ir-doped 1H-MoS_2_.

For Ir-doping, the DOS are very similar to the cases of Rh-doping, since the Ir atom belongs to the same column of the periodic table and has the same valence electron configuration as Rh. The difference is that the structure Mo_15_IrS_32_ is unambiguously half-metallic and can be utilized as a spin filter. Similarly, all structures possess a 1*μ*_B_ net magnetic moment (Fig. 14).

**Fig. 15:**
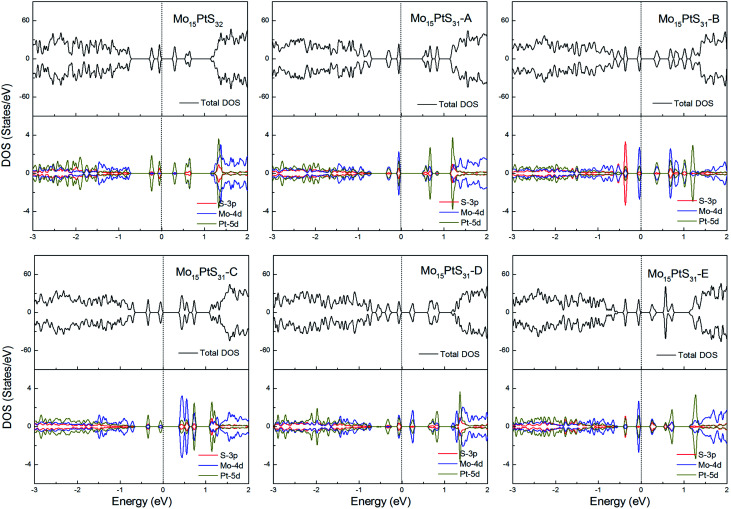
Total DOS and partial DOS for each atomic species in the all Pt-doped 1H-MoS_2_.

For the Pt-doped cases, all of the structures are nonmagnetic semiconductors, *i.e.*, they all have symmetrical spin-up and spin-down channels, and generate a series of energy gaps of 0.32, 0.58, 0.36, 0.45, 0.27, and 0.28 eV corresponding to Mo_15_PtS_32_, Mo_15_PtS_31_-A, Mo_15_PtS_31_-B, Mo_15_PtS_31_-C, Mo_15_PtS_31_-D, and Mo_15_PtS_31_-E, respectively. From the DOS figures, we can find that the d bands of the Pt ion mainly appear above the Fermi level and the strong hybridization of the S 3p and Pt 4d orbital, and the electrons tend to occupy the delocalized defective bands that are caused by the S vacancy (Fig. 15).

## Conclusion

In summary, based on first-principles calculations, we have investigated the effect of co-doping of G8 atoms and an S vacancy on the magnetic and electronic properties of monolayer MoS_2_. All of the mono G8-doped systems have magnetism, except for the Ni, Pd and Pt cases. According to the formation energy calculation, it is found that the nearest neighboring configurations for every doped system are preferred, and have lower formation energy (about 1.50 eV) than the remaining four configurations. Moreover, from the magnetic calculations, we find that the magnetic moment from the co-doping of Fe and an S vacancy (Fe-V_S_) is 2*μ*_B_, except for the nearest neighboring configuration, which is a nonmagnetic semiconductor. For the Co-V_S_, the total magnetic moments are 1 or 3*μ*_B_. Particularly for the Ni-V_S_ case, all of the doped models are nonmagnetic semiconductors. The high magnetic order structures with a net magnetic moment of 4*μ*_B_ are transition states that are about 200 meV above the ground state. In the case of Ru-V_S_, only the structure without an S vacancy has a net magnetic moment. For Os-V_S_, the Mo_15_OsS_31_ and Mo_15_OsS_31_-C configurations have a net magnetic moment of 2*μ*_B_. For Rh-V_S_ and Ir-V_S_, all of the configurations are magnetic and the total magnetic moments are 1*μ*_B_. Moreover, Ir-doped pristine 1H-MoS_2_ is a half-metal. In the case of Pd-V_S_, most configurations are nonmagnetic semiconductors except for the second-nearest neighboring configuration, which possesses a 2*μ*_B_ magnetic moment. For Pt-V_S_, all of the models are nonmagnetic semiconductors. Furthermore, the Curie temperatures (*T*_C_) are calculated within mean-field approximation. Our theoretical results show that Fe, Co, Ru, Rh, Os and Ir-doped MoS_2_ monolayers with particular distributions exhibit room-temperature ferromagnetism.

The magnetic properties of the co-doped system depend on the competition between the Hund coupling and the ligand field splitting. Our results suggest that the co-doping of G8 atoms and S vacancies is an efficient way to modulate the magnetic properties. The main obstacle is the control of the distribution. Our further work will focus on methods to decrease the formation energy of the defects and the maintenance of the magnetic moment.

## Conflicts of interest

There are no conflicts to declare.

## Supplementary Material

RA-008-C8RA01644E-s001
