# Supplementary material for: Modulating the magnetic properties of MoS2 monolayers by group VIII doping and vacancy engineering
Source: RSC Adv. 2018 May 23;8(34):18837–50. doi: 10.1039/c8ra01644e (PMC9080634; doi:10.1039/c8ra01644e)
Supplement: RA-008-C8RA01644E-s001 [file RA-008-C8RA01644E-s001.pdf]

Electronic Supplementary Material (ESI) for RSC Advances.
This journal is © The Royal Society of Chemistry 2018

Supporting Information

Table. S1 The magnetic moments with corresponding U values (in μB).

	$Fe_{U=}$ 4eV	$Co_{U=}$ 4eV	$Ni_{U=}$ 4eV	$Ru_{U=}$ 3eV	Rh_{U} =3.4eV	$Pd_{U=}$ 3.9eV	$Os_{U=}$ 2.7eV	$Ir_{U=}$ 2.8eV	$Pt_{U=}$ 2.9eV
$Mo_{15}XS_{32}$	2	3	0	2	1	0	2	1	0
$Mo_{15}XS_{31}$-A	0	1	0	0	1	0	0	1	0
$Mo_{15}XS_{31}$-B	2	3	0	0	1	2	0	1	0
$Mo_{15}XS_{31}$-C	2	1	0	0	1	0	2	1	0
$Mo_{15}XS_{31}$-D	2	1	0	0	1	0	0	1	0
$Mo_{15}XS_{31}$-E	2	3	0	0	1	0	0	1	0

Table. S2 The energies of non-magnetic states ($E_{non-magnetic}$ in eV) and magnetic states ($E_{magnetic}$ in eV) of Ni-doped special structure in DFT-PBE and PBE+U.

	DFT-PBE		$PBE+U_{U=4eV}$	
	$E_{non-magnetic}$	$E_{magnetic}(M=4\mu_B)$	$E_{non-magnetic}$	$E_{magnetic}(M=4\mu_B)$
$Mo_{15}NiS_{32}$	-337.325	-337.126	-335.101	-334.951
$Mo_{15}NiS_{31}$-B	-330.765	-330.424	-328.466	-328.238
$Mo_{15}NiS_{31}$-C	-330.949	-330.594	-328.672	-328.427
$Mo_{15}NiS_{31}$-E	-330.633	-330.425	-328.406	-328.244
